# 

**Published:** 1884-09-20

**Authors:** 


### THE SOUTHERN MEDICAL RECORD:

### A MONTHLY JOURNAL OF PRACTICAL MEDICINE.

**Terms: $2.00 per Annum, in Advance; 4 Copies $6.00; Single Copies 20 Cents.**

***"QUICQUID PRÆCIPIES ESTO BREVIS."***

### TABLE OF CONTENTS.

ORIGINAL AND SELECTED ARTICLES:

Nocturnal Terrors in Children, translated for the SOUTHERN MEDICAL RECORD from the Paris Journal de Medecine, by E. van Goidtsnoven, M.D., of Georgia, 321. A Distended Bladder Mistaken for a Possible Ovarian Tumor, 323. Intestinal Indigestion, 326. The Best Method of Disposal of the Dead, 329. Pathological Specimen—Extirpation of a Thyroid Gland weighing Sixteen and One-half Ounces—Recovery, 332. Spermatorrhœa—Impotence—Cure, 334. Ipecacuanha in Large Non-Emetic Dose in the Treatment of Cholera, 336. Report of the Illinois State Board of Health, 337.

ABSTRACTS AND GLEANINGS:

Summer Complaint in Children, 338. A Year's Experience in Tracheotomy, 340. Burns and Scalds; The Water-Cure in Diphtheria; Syrupus Acidi Hydriodici, 342. Lung Extirpation; Cases of Collapse and Sinking; Success under Difficulties, 343. Cardiac Dropsy; Cho'era, 344. Two Signs of True Convalescence in Enteric Fever; Phenic Acid in Yellow Fever; An Instant Remedy for Poisoning, 345. Nux Vomica as a Galactagogue; The Thermo-Cautery in the Treatment of Anal Fistule; Our Growing Longevity, 346. Flatulence, 347. Blindness from Ophthalmia Neonatorum; Oleate of Chloral Comp. for Pruritus; Death from Chlorate of Potassium, 348. Doctors in the West; The Diagnosis of Sciatica, 349. Erigeron Canadensis; Cholera and the Migration of Birds; The Song of the Tubercle Bacillus, 350.

SCIENTIFIC ITEMS:

Deep-Sea Curiosities; About Salt, 351. Watery Vapor in the Air; Limits of Telephonic Action, 352.

PRACTICAL NOTES AND FORMULÆ.

Burn Ointment Unexcelled; Painless Escharotics; Ringworm, 353. Hall's Solution of Strychnia; Fruit-Preserving Compound; Stye, 354. Cholera Mixtures, 355. Phthisis; Treatment of Chronic Adenitis; For Catarrh, 356.

EDITORIALS AND MISCELLANEOUS.

Editorial Notices; Resignation, 357. Death of Dr. W. G. Drake; Monteagle as a Summer Resort; Medical Literature in the South, 358. Books and Pamphlets Received, 359. Receipted; Special Notices, 360.

